# 4D structural changes and pore network model of biomass during pyrolysis

**DOI:** 10.1038/s41598-023-49919-z

**Published:** 2023-12-21

**Authors:** Ifeoma Gloria Edeh, Ondrej Masek, Florian Fusseis

**Affiliations:** 1 UK Biochar Research Centre, School of Geosciences, University of Edinburgh, Edinburgh, UK; 2 Division of Earth Sciences and Geography, RWTH Aachen University, Aachen, Germany

**Keywords:** Climate sciences, Environmental sciences, Hydrology, Materials science

## Abstract

Biochar is an engineered carbon-rich substance used for soil improvement, environmental management, and other diverse applications. To date, the understanding of how biomass affects biochar microstructure has been limited due to the complexity of analysis involved in tracing the changes in the physical structure of biomass as it undergoes thermochemical conversion. In this study, we used synchrotron x-ray micro-tomography to visualize changes in the internal structure of biochar from diverse feedstock (miscanthus straw pellets, wheat straw pellets, oilseed rape straw pellets, and rice husk) during pyrolysis by collecting a sequence of 3D scans at 50 °C intervals during progressive heating from 50 °C to 800 °C. The results show a strong dependence of biochar porosity on feedstock as well as pyrolysis temperature, with observed porosity in the range of 7.41–60.56%. Our results show that the porosity, total surface area, pore volume, and equivalent diameter of the largest pore increases with increasing pyrolysis temperature up to about 550 °C. The most dramatic development of pore structure occurred in the temperature range of 350–450 °C. This understanding is pivotal for optimizing biochar’s properties for specific applications in soil improvement, environmental management, and beyond. By elucidating the nuanced variations in biochar’s physical characteristics across different production temperatures and feedstocks, this research advances the practical application of biochar, offering significant benefits in agricultural, environmental, and engineering contexts.

## Introduction

Biochar is a carbon-rich residue of biomass pyrolysis, i.e. heating of organic materials to a high temperature in the absence of oxygen. Although the main purpose of biochar is to remove and safely store atmospheric carbon, it has a range of other applications. It has the potential to increase carbon sequestration^1^, improve soil properties^2–4^, increase crop yield^5^, and reduce pollutants in soils^6^. The efficiency of biochar in these applications depends on its physical and chemical properties which vary greatly depending on the feedstock and production conditions used. The pore structural properties of biochar (porosity, pore size distribution, surface area etc.) are among the most important properties determining its efficacy^7^. Many ecological functions of biochar are determined by its pore structural properties. For example, when added to soils, biochar intrapores help increase soil water retention^8,9^, and biochar porosity and pore size distribution help improve soil physical properties and plant growth^10,11^. Biochar pore spaces can also serve as a habitat for microorganisms^12,13^. To select or engineer biochar for specific applications, it is critical to understand the pore structural properties of biochar and how it relates to the porosity of starting material and how it transforms during processing.

X-ray computed microtomography (XµCT) is a non-destructive, radiographic imaging technique used to produce 3D images that can provide detailed information on various internal attributes of an object^14^.One limitation of XµCT using conventional x-ray sources is that only samples with either adequate x-ray attenuation coefficients or composites with sufficient difference between single coefficients could be used. The use of synchrotron light sources, which produce a tuneable, partially coherent x-ray beam of extreme brightness, makes it possible to acquire phase contrast images showing samples attributes even to submicron levels^15,16,^^17^. High-resolution 3D images obtained by synchrotron x-ray XµCT (SXµCT) provide useful information about the changes in biomass undergoing thermochemical decomposition at elevated temperatures, and the microstructural properties of biochar produced from different feedstock and at different ranges of pyrolysis temperatures. Further, the short acquisition times at synchrotron light sources enable time-resolved (i.e. 4D) studies^17^.

In biochar studies, XµCT has been used to investigate the internal pore structure, characterise porosity, pore size distribution, specific surface area and structural anisotropy of biochar produced from different feedstock materials and under varying pyrolysis conditions^18–23^. These studies measured biochar pore characteristics of a set of discrete samples, each pyrolyzed at specific conditions. This has limitations in terms of interpretation of the results, as changes in the structure cannot be assessed directly, because the samples observed are not identical. No study to date has achieved a comprehensive 4D SXµCT-based characterization of the development of microstructural properties of different biomass and biochar through the full pyrolysis temperature range using a single sample. Unlike the discrete sample approach, this process allows for direct measurement of accurate changes that occur in biomass and biochar with increasing pyrolysis temperature, such as shrinkage or growth of pores in biomass, their interconnection, or blockages, as well as the formation of cracks. Such data is indispensable for an in-depth understanding of biochar development, and therefore for designing engineered biochar, with properties tuned to its intended applications. It is also an important input to the development and validation of biomass pyrolysis models that have to date not considered biomass porosity and its changes, or only in simplistic ways^24,25^.

In this study, SXµCT was used to visualize the internal structure of biomass particles undergoing pyrolysis, to provide new and extensive insights into 4D structural changes of biochar produced from different feedstock and under different temperatures. This allows for accurate observation of the microstructural changes that occur due to feedstock composition, biomass cellular structure, and pyrolysis temperatures. The results from this study are not only important for the use of biochar as soil amendments but also for all other applications relying on biochar pore characteristics.

## Results and discussion

### Visualization of the evolving pore structures

**Figure 1:**
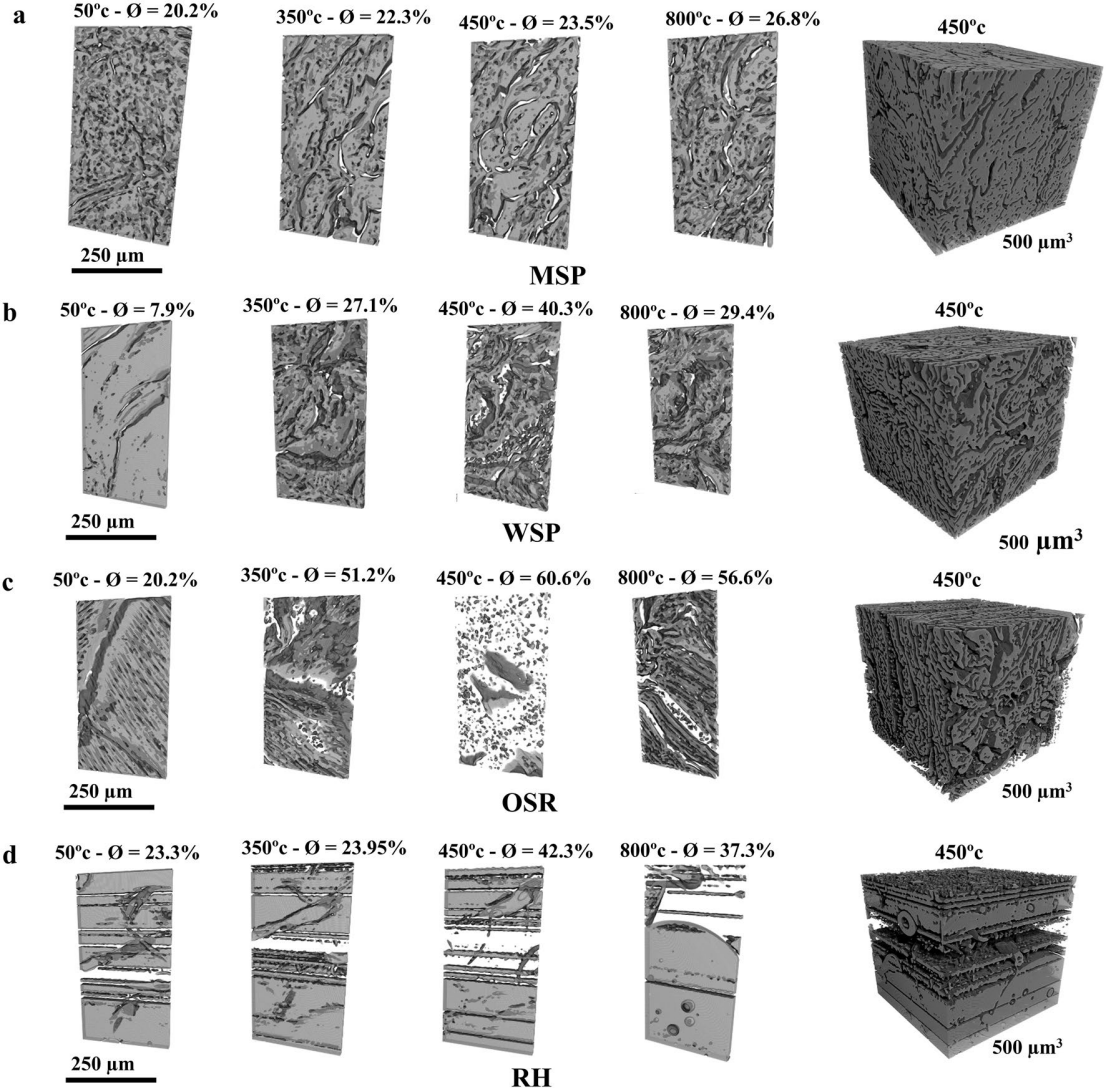
3D pore morphological view of biochar and porosity (ø) produced from (**a**) miscanthus straw pellet (MSP), (**b**) wheat straw pellet (WSP), (**c**) oilseed rape straw pellet (OSR), and (**d**) rice husk (RH) at significant pyrolysis temperatures (complete pyrolysis temperatures are provided as supplementary data (Figs. S1 and S2).

Visualization of the imaged samples in grayscale are shown in Fig. 1a–d. The 3D view of the representative elementary volume (REV) and a subsample view of 25 × 250 × 500 µm were used to show the pore morphology of all imaged samples (Supplementary Figs. S1, S2). Visual assessment of the images shows that biochar samples exhibit a lot of variations in the pore morphology. As expected, variations were observed for the different feedstocks and with changing pyrolysis temperatures. In general, miscanthus straw pellets (MSP), wheat straw pellets (WSP) and oilseed rape straw pellets (OSR) consist of a minimal number of lateral pores, while rice husk (RH) consist of more lateral-oriented pores. Pores formed in biochar produced from MSP and WSP are visually more connected than those of OSR and RH. Modification of the biomass before pyrolysis had a strong effect on the pore morphology of the biochar produced. Pelletization, which entails compressing small biomass particles at high pressure and elevated temperature into uniform cylindrical pellets, had a significant effect on the biochar pore morphology. Biochar produced from pelleted biomass had a more compact structure and similar visual morphology (Fig. 1a–d).

**Figure 2:**
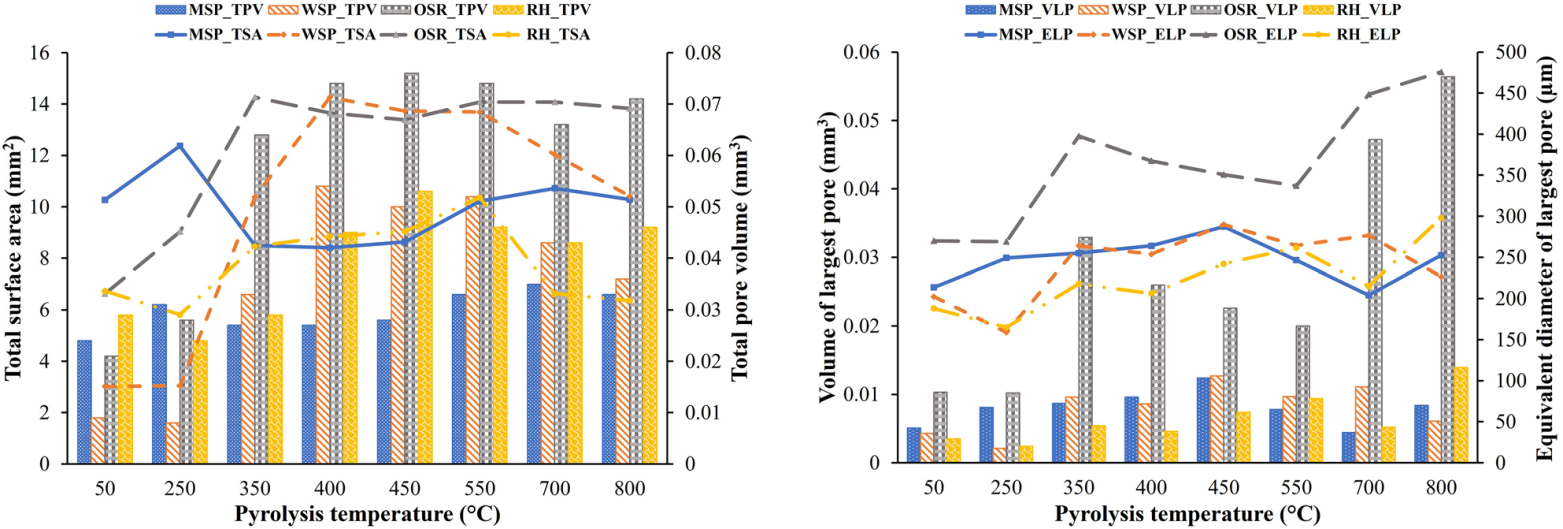
3D pore structural analysis of biochar produced from different feedstock and pyrolysis temperatures.

**Figure 3:**
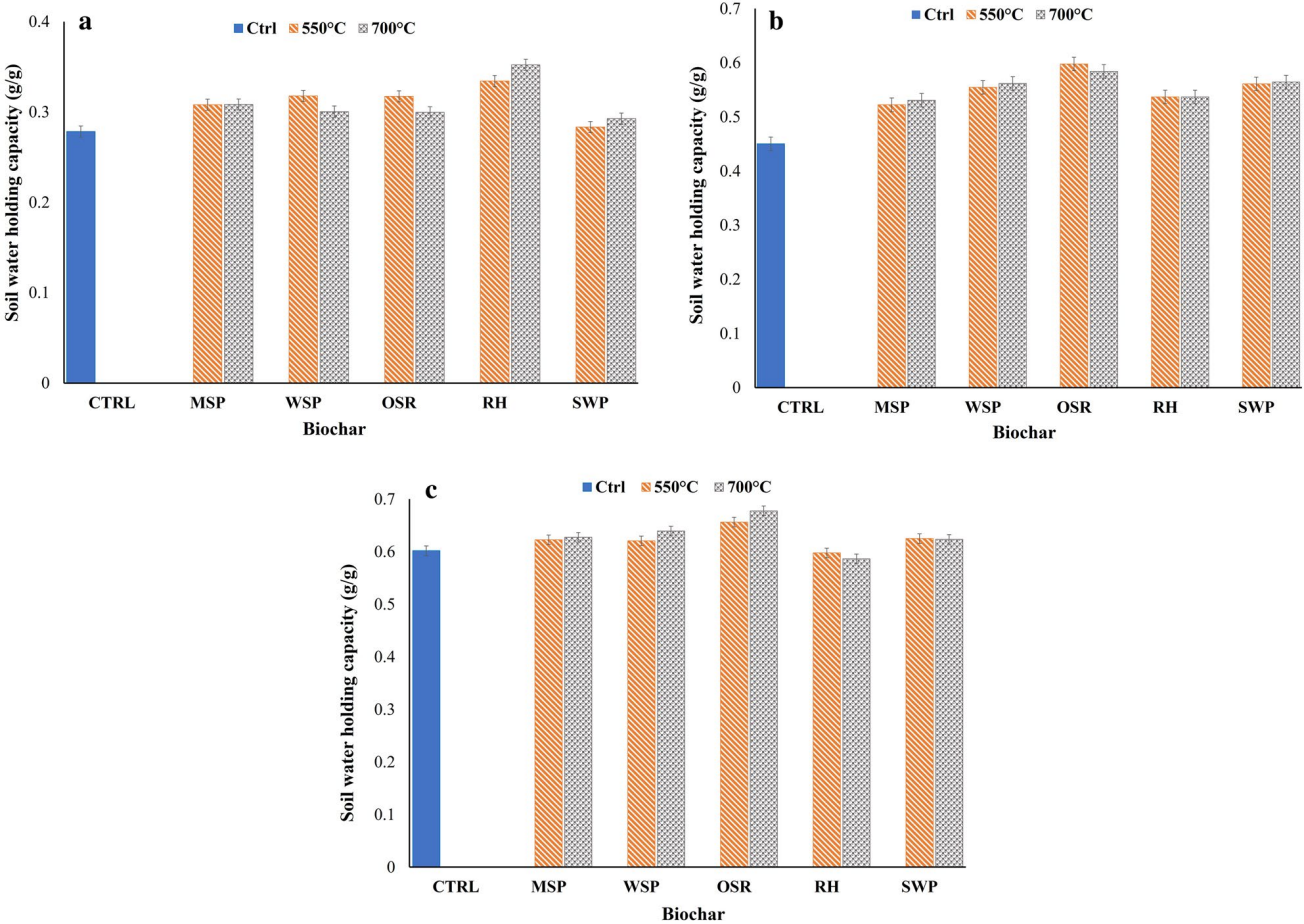
Effect of different biochar on soil water holding capacity of (**a**) sandy loam, (**b**) loamy soil and (**c**) clay soil. Error bars represent the standard error.

The pore structural analysis results as shown in the two graphs in Fig. 2 reveals that, apart from the total surface area, higher values for porosity, total pore volume, and pore volume and equivalent diameter of the largest pore (VELP) were obtained for biochar produced from OSR, followed by WSP and RH. This is attributed to the lower cellulose content of their biomass compared to that of MSP (Table 1). The unique nature of the lignocellulosic properties of the biomass used is one of the most important components in determining the characteristics of the biochar produced^26^. Typically, the lignocellulosic composition of biomass is made up of 3 main building blocks: cellulose, hemicellulose, and lignin. During pyrolysis of biomass to produce biochar, biomass with higher cellulose content and lower lignin content normally produces biochar with lower porosity^7,27, 28^. This is due to the stability of lignin at higher temperatures compared to cellulose and hemicellulose which would lead to preservation of its pore structure. It can be concluded that biochar produced from OSR should be desirable for applications needing higher porosity values such as increased soil water retention. This prediction was validated through a laboratory experiment (detail in Sect. “Laboratory experiment”) looking at the effect of biochar on the soil water holding capacity of three different soil types (Fig. 3). The results showed that biochar from OSR significantly increased water holding capacity compared to other feedstocks for loamy and clay soil types.

**Table 1:** Summary of the lignocellulosic composition of biomass feedstock (% dry matter).

Feedstock	Cellulose	Hemicellulose	Total lignin	Ash
Miscanthus straw pellet	37.96 ± 0.01	19.86 ± 0.19	19.43 ± 0.01	4.97 ± 0.05
Wheat straw pellet	34.86 ± 0.46	20.52 ± 0.02	18.99 ± 0.23	6.32 ± 0.04
Oilseed rape straw	29.70 ± 0.51	14.67 ± 0.19	17.81 ± 0.20	6.44 ± 0.18
Rice husk	30.60 ± 0.23	12.49 ± 0.17	20.78 ± 0.28	24.50 ± 0.19

The total surface area (TSA) of the studied biochar follows a different trend from that of the porosity. Although the highest TSA is found at OSR350, the TSA of biochar produced from MSP is higher than that of RH biochars. This could be attributed to the higher ash content of RH biomass (> 70% higher, see Table 1). During pyrolysis of biomass, partial filling, or blockage of micropores with minerals (ash) can result in lower surface area^29^. The higher proportion of micropores in MSP with respect to other feedstocks (Fig. 5), could explain its higher TSA even though it had lower total porosity. Generally, the smaller the pores of the biochar, the higher the surface area^7^. Although biochar produced from MSP had lower porosity (below 30%), it had comparably higher total surface area than some of the other biochars which could led to increased availability of surface sites for increased cation exchange, microbial colonization, and nutrient fixation in soils compared to biochar with lower surface area^30–32^. As a result of methodological differences, it is difficult to accurately compare the observed pore characteristics to those previously reported in the literature. Surface area calculated from SXµCT images cannot be directly compared to that obtained using gas adsorption methods like the Brunauer–Emmett–Teller (BET) Method because surface area as determined by SXµCT is heavily influenced by image resolution, and cannot resolve sub-micron pores. Using Alkali-Silica Reaction, Oesch, et al.^33^ reported an unclear relationship between the measured BET and XµCT values for both the external granule surface and the surface of externally accessible pores and cracks. This was attributed to the large difference in the sensitive size ranges for BET and XµCT. Due to the poor spatial resolution of XµCT, Oesch et al.^33^ discovered that the BET-measured specific surface areas are approximately three orders of magnitude greater than the XµCT-measured values. This suggests that BET may be more beneficial for analysing surface area.

Besides biomass structure and composition, pyrolysis conditions (especially peak temperature), are other key factors affecting biochar pore morphology. In our experiments increasing pyrolysis temperature led to the development of larger and more elongated slit-shaped pores throughout the biochar structures for MSP, WSP and OSR biochar, while for RH, the lateral pores become bigger, and more lateral pores formed with increased temperature (Fig. 1a–d). Overall, an increase in porosity, TSA, total pore volume, and VELP were observed with increasing pyrolysis temperature up to about 400–450 °C for all feedstocks. Many studies have reported an increase in the surface area, porosity and pore volume of biochar with increasing pyrolysis temperature^7,23, 28, 34–36^. In general, a rising pyrolysis temperature leads to the elimination of more volatiles and the condensation of amorphous carbon into crystalline carbon. This results in cracks and the creation of sparse regions in the biomass, which contributes to the formation of more pores^37^. The observed development of biochar pore structure with increasing temperature could also be due to the degradation of organic materials (cellulose, hemicellulose, and lignin), which leads to the formation of further pores^38^. An increase in surface area particularly with increasing pyrolysis temperature is related to the degradation of aliphatic alkyl and ester groups^7,23, 28, 34–36, 39^.

The most remarkable development of porosity in the observable pore size range happens at lower pyrolysis temperatures (350–450 °C). Above 550 °C, the porosity of most of the biochar samples began to decrease. This is because with increasing temperatures above 450 °C, the relative ash content of biochar increases, with the progress of devolatilization of organic matter. This slows down the development of pores and surface area^40^. This phenomenon of reduced pore structural development above temperatures of 400–450 °C has also been observed by other studies^41–43^.

### Pore size distribution and categorization

**Figure 4:**
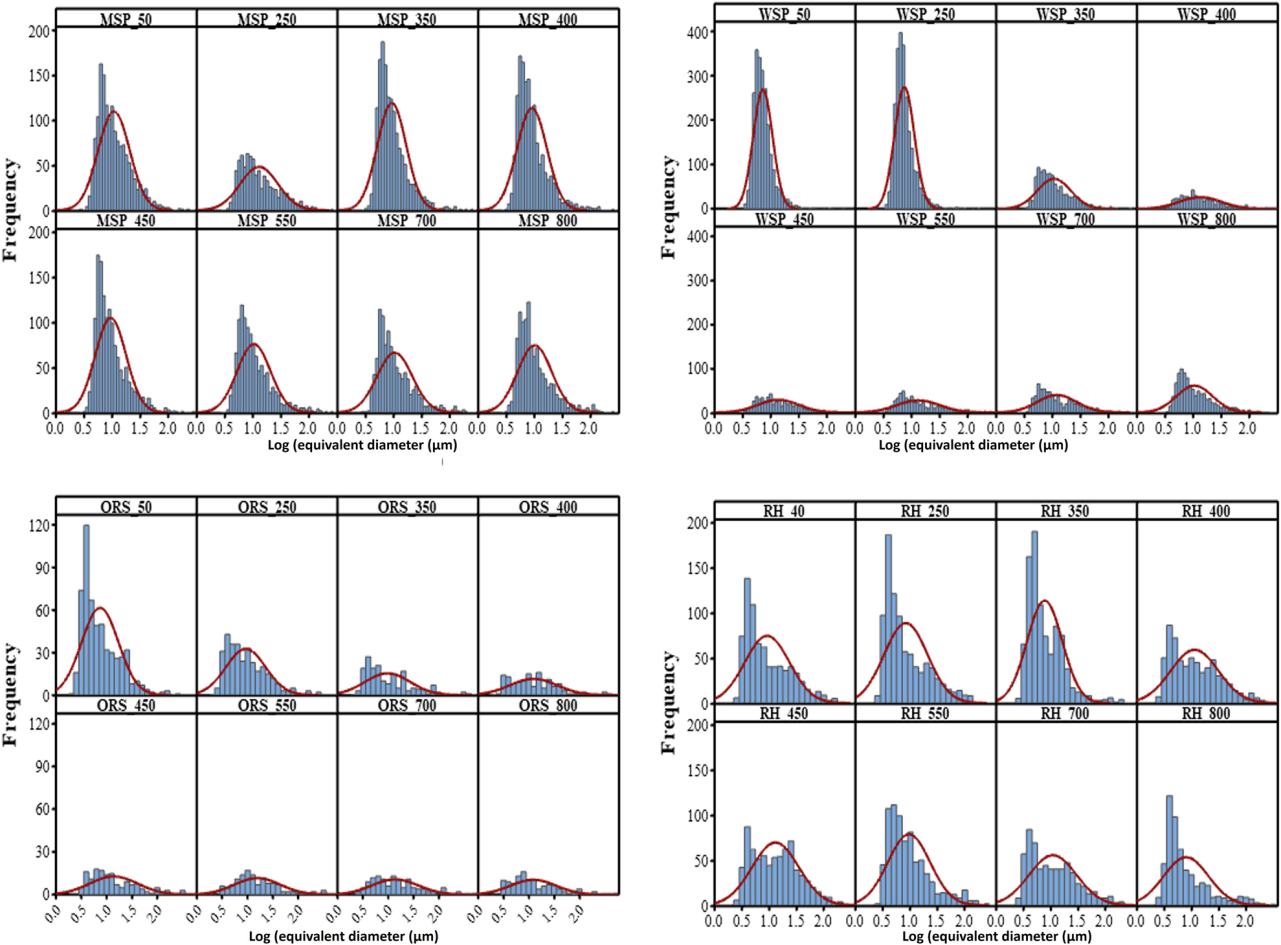
Pore size distribution of biochar produced from miscanthus straw pellet (MSP), wheat straw pellet (WSP), oilseed rape straw pellet (OSR), and rice husk (RH) at different pyrolysis temperatures.

**Figure 5:**
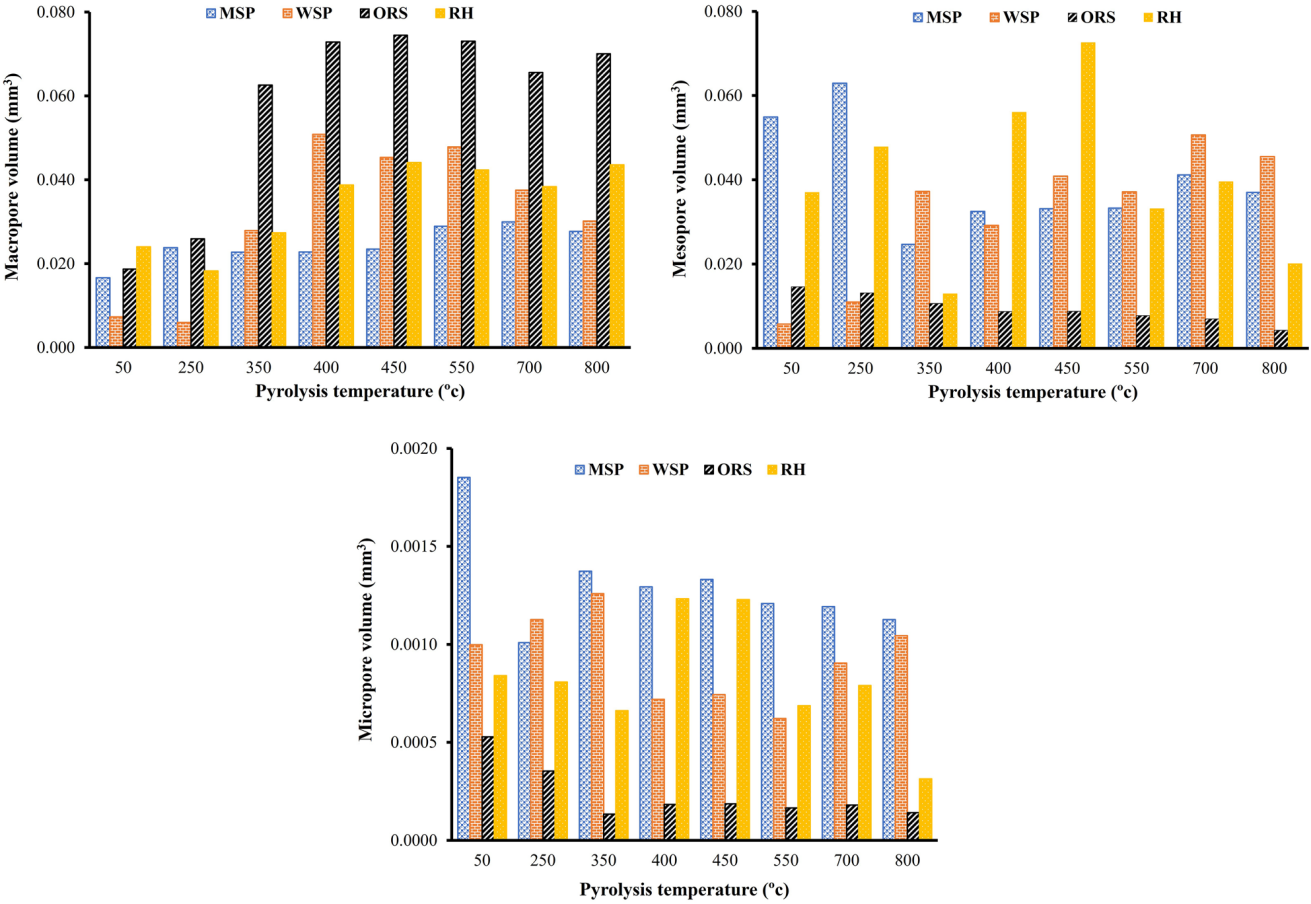
Development of macropore, mesopore and micropore volumes of biochar produced from miscanthus straw pellet (MSP), wheat straw pellet (WSP), oilseed rape straw pellet (OSR), and rice husk (RH) and at different pyrolysis temperatures.

Pore size distributions and pore categorisation of the imaged samples are shown in Figs. 4 and 5. On the basis of these data, pores were grouped into macropores (> 75 µm), mesopores (30–75 µm) and micropores (5–30 µm), and their volumes determined (Fig. 5). The properties of feedstock biomass appear to be a critical component in shaping the biochar pore size distribution, even when the native porosity is altered by mechanical pre-processing, such as pelletization. Biochars produced from OSR and WSP have greater macropore volumes while biochars produced from RH and MSP have greater mesopore and micropore volumes, respectively. The distribution of the biochar pores can influence how these biochars are utilized. Biochar produced from OSR at pyrolysis temperatures > 350° would be desirable for increasing hydraulic conductivity in soils and could serve as a suitable habitat for protists and nematodes due to their greater macropore volumes^8^. Biochars produced from MSP, WSP and RH at a temperature below 550 °C would be most beneficial for increasing available plant water content because of their higher mesopore volume, while biochars produced from MSP and WSP at lower pyrolysis temperature (< 350 °C) could provide a habitat for many bacteria, and fungal hyphae due to their greater micropore volume^44^.

Pyrolysis temperature played a role in creating variations in the pore size distribution of the biochar. Generally, at temperatures below 550 °C the macropore and mesopore increased with increasing temperature, while the micropore decreased (Fig. 5). This could be due to the elimination of volatiles at higher temperatures leading to the formation of larger pores^28^ as well as the merging of micropores to form mesopores. The overall frequency of pore sizes reduces with increasing pyrolysis temperature (Fig. 4), indicating a coalescing of pores into fewer larger pores. At lower temperatures, there were clearly more pores in the range of 3–10 µm. With an increase in temperature, the distribution of pores became more uniform, especially for WSP and OSR at temperatures greater than 350 °C.

### Pore network model (PNM) and pore connectivity

The visualization of the PNM for all biochar produced is shown in Supplementary Figs. S3, S4. The PNM is a network of pores and throats. The spheres represent pores scaled with the equivalent diameter (pore size) and the lines represent the pore throat scaled with the throat length^45^. Clear differences in PNM exist among the biochar produced from different feedstock and at different pyrolysis temperatures.

**Figure 6:**
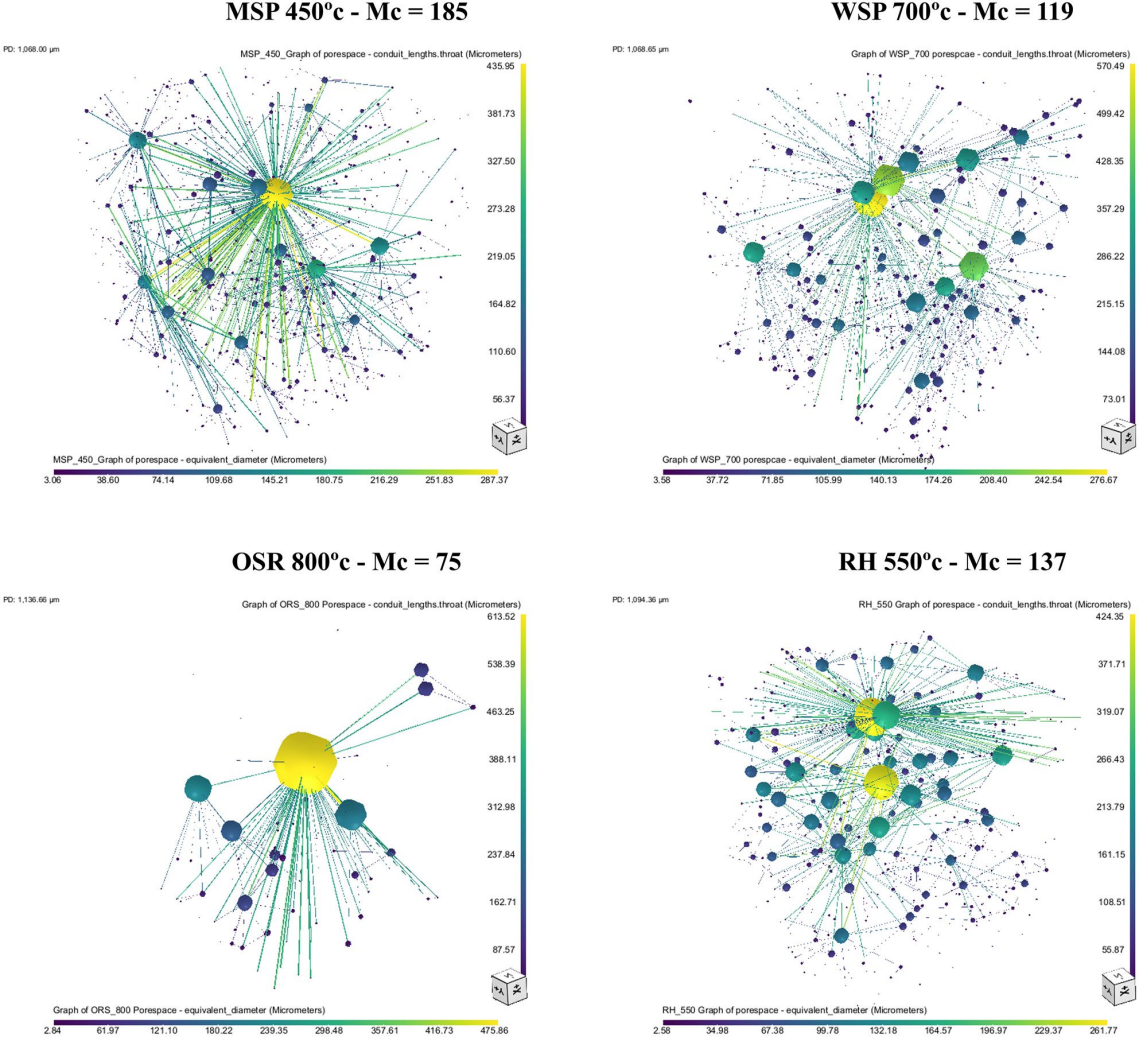
3D pore network model of biochar produced from miscanthus straw pellet (MSP), wheat straw pellet (WSP), oilseed rape straw pellet (OSR), and rice husk (RH) at highest maximum connectivity (Mc) (complete pyrolysis temperatures are provided as supplementary data, Figs. S3 and S4).

The PNM of biochar made from OSR stood out among the studied materials, with lower pore density, fewer throats, and wider pores (Fig. 6). While the PNMs of biochar made from MSP, WSP, and RH all have a dense network of pores and throats. This is due to its larger macropores in OSR, and a smaller number of pores (see pore size distribution data, Fig. 6). Most of the throats of biochar produced from OSR are connected to the longest interconnected pores. The pore connectivity impacts water flow and movement and determines whether water entrapment would occur during the draining process, or air entrapment during wetting towards saturation. Large pores that run the length of the biochar, as seen in OSR biochars, can dominate water movement. On the other hand, if the pores are not connected to one another, there will be no flow at all^46^.

**Figure 7:**
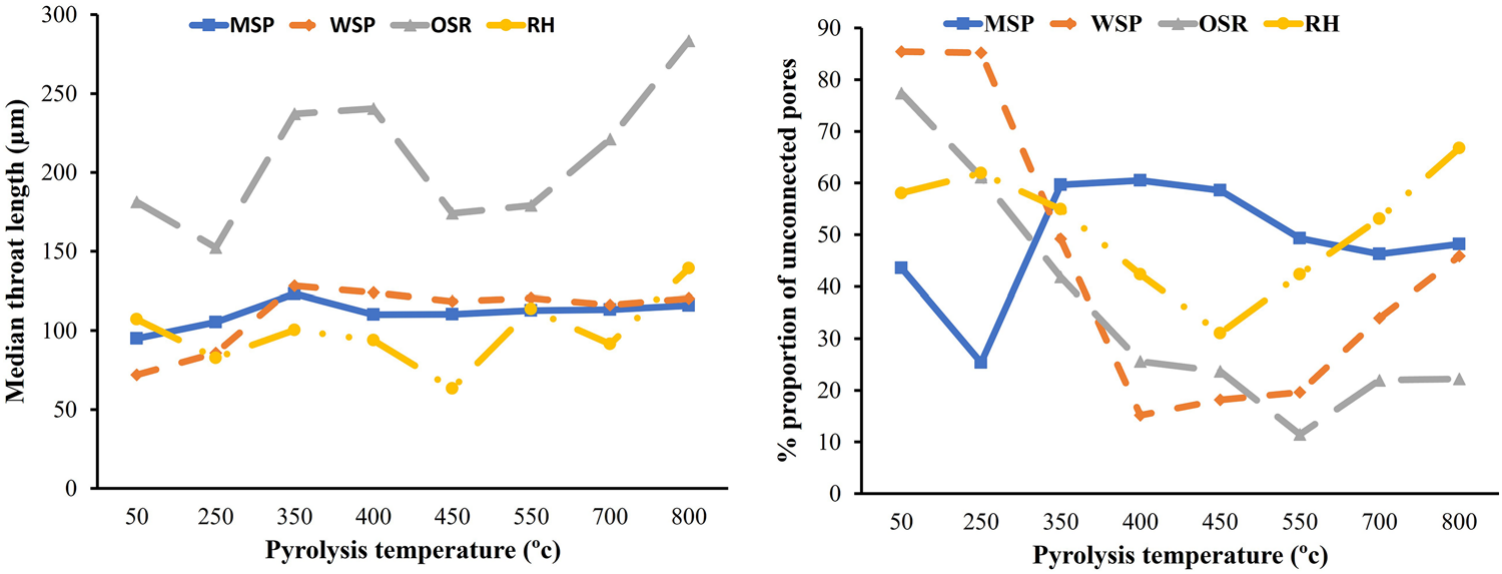
Connectivity parameters of biochar produced from miscanthus straw pellet (MSP), wheat straw pellet (WSP), oilseed rape straw pellet (OSR), and rice husk (RH)and at different pyrolysis temperatures.

We compared the connectivity parameters, the proportion of unconnected pores (PUP), and the median throat length of all the biochar produced (Fig. 7). Both varied depending on the feedstock type. At lower pyrolysis temperature (< 350 °C), pores of RH and MSP were more connected (PUP < 60%) than those of OSR and WSP (PUP > 60%), while at higher pyrolysis temperature (> 400 °C), the pores of OSR and WSP were more connected (PUP < 30%). With increasing temperature, there was a significant decrease in the PUP of biochar produced from OSR and WSP (PUP from 77.36–11.45%, and 85.4–15.2%, respectively) while the changes in the PUP of biochar from MSP and RH were not so drastic. This could be related to the difference in lignin content; OSR and WSP had lower lignin content compared to MSP and RH. Among the three lignocellulosic components, lignin is the most thermally stable^28^. The gradual change in pore connectivity of MSP and RH could be attributed to the preservation of its pore structure by the stability of lignin.

The changes in pore connectivity with increasing pyrolysis temperatures were feedstock dependent, and hint at the importance of the lignocellulosic composition as well as the content and composition of the mineral matter in biomass. As a general trend, there was a reduction in pore connectivity at temperatures above 550 °C for all biomass feedstocks. This could be related to enhanced lignin decomposition at higher temperatures^47^. This degradation may result in pore restructuring and subsequent reduction in pore connectivity.

In conclusion, we present the first use of 4D SXµCT for biochar pore characterization. The 4D image visualization and analysis of data obtained from SXµCT allowed for a better understanding of the factors contributing to the pore development of biochar. Our results showed that the type of feedstock biomass and pyrolysis temperature played a significant role in how the biochar pore structure evolved. Both pyrolysis temperature and feedstock biomass largely affected the porosity, total surface area, total pore volume, pore volume and equivalent diameter of longest interconnected pore, and pore size distribution. The effect of pyrolysis temperature on pore connectivity was dependent on the feedstock; however, for all biomass feedstock, there was a reduction in pore connectivity when pyrolysis temperature exceeded 550 °C. Biochar produced from OSR would be desirable for increasing hydraulic conductivity in soils and can serve as a habitat for protists and nematodes due to its greater porosity and higher macropore volumes. In contrast, biochar produced from MSP and WSP (< 350 °C) would be desirable to provide a habitat for many bacterial and fungal hyphae due to their greater micropore volume. Of all feedstocks tested, biochar produced from RH, MSP and WSP would be most beneficial for increasing available plant water content because of their higher mesopore volume. Choosing the right feedstock biomass and production condition could help produce optimized biochar for specific environmental functions.

## Methodology

### Biomass feedstock

Four types of biomass feedstock were selected for pyrolysis (MSP, WSP, ORS, and RH). For information on where biomass was sourced and the proximate analysis results of each feedstock, see Ref.^48,49^. Apart from rice husk, all other biomasses were pelleted to improve handling and processing in the pyrolysis unit. The lignocellulose compositions of the biomasses were determined at an external laboratory (Celignis Analytical) via wet-chemistry analysis^50^. Average values and standard deviation for each compositional data are shown in Table 1.

### Experimental setup, pyrolysis, and image acquisition via synchrotron x-ray microtomography

The experiment was conducted at the 2-BM beamline of the Advanced Photon Source (APS) at the Argonne National Laboratory, IL, USA, where 3-dimensional images of the samples were obtained using synchrotron XµCT (SXµCT). There are several advantages associated with using synchrotron radiation over conventional x-ray sources. The high-quality monochromatic x-ray beam significantly improves image quality, and it allows for fast data acquisition at very high spatial resolution producing accurate mapping of the internal structure of samples^17,51^.

**Figure 8:**
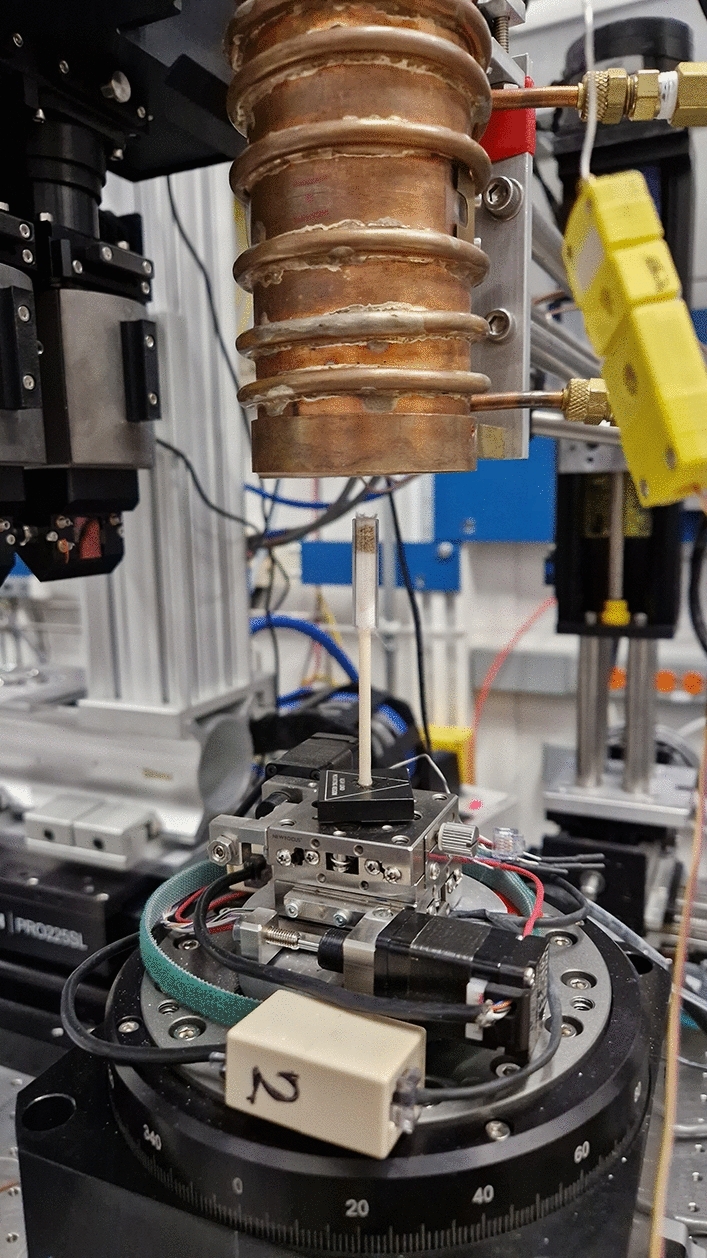
Experimental setup at the advanced photon source, USA.

The principles and method of data collection with SXµCT has been widely discussed^17,52, 53^. The experimental setup at the microtomography beam line 2BM at the Advanced Photon Source (USA) included a sample holder that allowed controlling the atmosphere around the sample. The holder was inserted into a small x-ray transparent furnace, which was mounted above the rotation stage (Fig. 8). A small subsample of the feedstock (weighing between 2.3 and 4.8 mg) was placed in the sample holder and heated in an N_2_ atmosphere at a rate of 7.5 °C/min from room temperature to 800 °C. Each sample was scanned at the following temperatures during heating; 50, 110, 250, 275, 300, 325, 350, 375, 400, 425, 450, 500, 550, 600, 700 and 800 °C (16 scans per sample) by a PCO Edge camera with a sensor size of 2560 × 2160 pixel with a 7.5 × magnification lens. During each scan, the sample was rotated from 0 to 180° at 18°.sec^–1^ leading to a total scan time of 10 s.

From the 2D radiographs collected during each scan, a 3D model was reconstructed using the reconstruction software Octopus version 8.9.4^54^. The final image stack for each scan had the following specifications: 2560 × 2160 pixels × 2025 slices, a voxel size of 0.87^3^ µm^3^, 32 bit depth and a size of 26.4 GB.

### Digital image processing

In this study, images of 8 different heating steps for each feedstock were analysed (50, 250, 350, 400, 450, 550, 700 and 800 °C) resulting in 32 datasets (Supplementary Table S1). The heating steps were selected based on preliminary analysis and correspond to stages in the pyrolysis process where major changes in the biomass and biochar structure occurred. All image analysis was done using a combination of the open-source program Fiji^55^ and Dragonfly (Dragonfly software, version 2020.2). The greyscale image histogram distributions of the SXµCT data show that all images in a time series had similar grey value distribution (Supplementary Fig. S5), which justifies similar image processing to be carried out for all image datasets.

To reduce processing time, the samples were cropped, and a representative elementary volume (REV) was selected. The REV is the smallest volume which represents the entire sample within a certain deviation in which measurements can be taken^56^. The procedure with which the REV for our samples were selected is provided in the supplementary material (Section S1). The REV of 500^3^ voxels was selected for all the datasets.

Image filtering to improve segmentation quality, denoise the image and increase the contrast between particle and pore spaces was done in Dragonfly by applying the 3D median filter with a kernel size of 5. The median filter smoothens images by replacing each pixel with the median value of its neighbours^57^. After testing different thresholding filters (see supplementary information—Section S2 -for details), Otsu thresholding^58^ was selected to produce a binary image (foreground and background) which was then used for the image analysis. Otsu threshold clustering algorithm searches for the threshold that minimizes the intra-class variance defined as a weighted sum of variances of the two classes^58^. To quantitatively measure pore characteristics, the thresholded 3D images were labelled utilizing the Open Pore Network Model (OPNM) plugin in Dragonfly^45^.

### Image analysis

Image analysis was done to determine the porosity, total surface area, total pore volume, and pore volume and equivalent diameter of the longest interconnected pore for all the imaged samples. The porosity is simply the fraction of pore volume in the image sample and was calculated as the number of voxels classified as pores divided by the number of voxels in the REV. The pore volume is the volume occupied by each labelled pore and the total pore volume was calculated as the sum of all the labelled pore volumes. The total surface area is the sum of the surface area of all labelled pores.

To calculate the pore size, we used the equivalent diameter of the pore, which is one of the methods for deriving pore sizes^59^. The equivalent diameter is calculated as $d=\frac{4A}{p}$. Where A is the area of the pore and p is the perimeter^60^. The pore size distribution was determined as a frequency distribution of the equivalent diameter. In relation to soil hydrology, the pore sizes were categorized into macropores (> 75 µm), mesopores (30–75 µm) and micropores (5–30 µm)^61^. The percentage volume of pores for each category was also obtained.

### Pore network model

A well-known method of simulating transport in porous media is pore network modelling^62,63^. After segmenting our 3D images into the binary image of pore and solid phase, the pore phase was extracted for pore network modelling. We used the Open Pore Network Model (OPNM) plugin in Dragonfly to generate the pore network model for each of the datasets^45^. The PNM in 3D shows spheres which represent pores scaled with pore size and lines which represents pore throats scaled with throat length. From this model, quantitative data were extracted for median throat length and percentage proportion of unconnected pores (PUP). Isolated pores were grouped as unconnected pore space and the percentage proportion was calculated as $\frac{\sum px}{pt}\times 100$, where px is the number of unconnected pores and pt is the total number of labelled pores.

### Laboratory experiment

A laboratory experiment was conducted to validate the effect of biochar porosity on soil water retention. Three soil types (sandy loam, loam, and clay soil) representing broadly 3 textural classes of soil (coarse, medium, and fine) were used for the experiment. These soils were obtained from the Landwirtschaftliche Untersuchungs und Forschungsanstalt (LUFA) at Speyer, Germany. Basic soil properties for the three soil types are provided in Table 2. The soil samples were dried and sieved with a 2 mm sieve.

**Table 2:** Soil properties (mean values of different batch analyses ± standard deviation.

Standard soil type no	2.2	2.4	6S
Organic carbon (%C)	1.71 ± 0.30	1.99 ± 0.21	1.78 ± 0.08
Nitrogen (% N)	0.18 ± 0.03	0.22 ± 0.01	0.18 ± 0.01
pH value (0.01 M CaCl_2_)	5.6 ± 0.4	7.4 ± 0.1	7.2 ± 0.1
Cation exchange capacity (meq/100 g)	9.2 ± 1.4	23.0 ± 14.9	24.2 ± 5.3
% Clay	8.0 ± 1.5	26.3 ± 0.5	40.9 ± 1.5
% Silt	13.7 ± 1.0	41.6 ± 1.5	35.3 ± 0.6
% Sand	78.3 ± 1.0	32.1 ± 1.6	23.8 ± 1.9
Soil texture	Sandy loam	Loam	Clay
Water holding capacity (g/100 g)	44.8 ± 2.9	44.8 ± 2.0	41.7 ± 1.0

All values refer to dry matter.

Four UKBRC standard biochars (RH, MSP, ORS, and WSP) pyrolyzed at 2 temperatures (550 and 700 °C were used for the experiment. The method of production and pyrolysis is described in Ref.^48^.

To prepare the soil-biochar mixtures, each biochar from the different biomasses were added to the 3 soil types maintain the same particle size range for all the biochar (2–0.5 mm) at 2% dry weight with 5 replications giving a total of 55 biochar treatments for each soil type. Following spraying of water to achieve approximately 15% gravimetric moisture content, the soil-biochar mixtures were incubated at 30 °C in sealed buckets for 10 days in a temperature-controlled environment with enough space for microaggregate formation. During incubation, water was added to the mixture twice to maintain moisture content at 15%. After 10 days, the soil-biochar mixtures were air-dried and then sieved over a 5 mm sieve. Soil water holding capacity were determined according to the method adopted by Verheijen et al.^64^. The soil-biochar mixtures and control sampled were packed in Polyvinyl Chloride (PVC) columns which were covered with nets at the bottom to allow for free drainage. The samples were saturated from bottom to top with water by placing the columns into a bucket filled with water. After saturation, the samples were allowed to drain freely for 24 h until no more drainage occurred. The drained samples were weighed, oven dried, and weighed again. The gravimetric soil water holding capacity (SWHC) was calculated as;

$$\mathrm{SWHC }= \frac{{W}_{d}- {W}_{od}}{{W}_{od}},$$

where W_d_ = weight of drained mixture and W_od_ = weight of oven dried mixture.

To evaluate the effect of biochar on soil water holding capacity, statistical comparison using one way analysis of variance and Fisher LSD test at 95% confidence were used to test for significant differences between means.

### Supplementary Information

Supplementary Information.

## Data Availability

The datasets generated during and/or analysed during the current study are available from the corresponding author (IGE) on reasonable request.
