# Supplementary material for: 4D structural changes and pore network model of biomass during pyrolysis
Source: Sci Rep. 2023 Dec 21;13:22863. doi: 10.1038/s41598-023-49919-z (PMC10739905; doi:10.1038/s41598-023-49919-z)
Supplement: Supplementary file 1 — Supplementary Information. [file 41598_2023_49919_MOESM1_ESM.docx]

**4D structural changes and pore network model of biomass during pyrolysis**

Ifeoma Gloria Edeh*, Ondrej Masek and Florian Fusseis

UK Biochar Research Centre, School of Geosciences, University of Edinburgh, UK.

*Corresponding author: [edeh.gloria@gmail.com](mailto:edeh.gloria@gmail.com); [Ifeoma.edeh@ed.ac.uk](mailto:Ifeoma.edeh@ed.ac.uk)

**Supplementary Material**


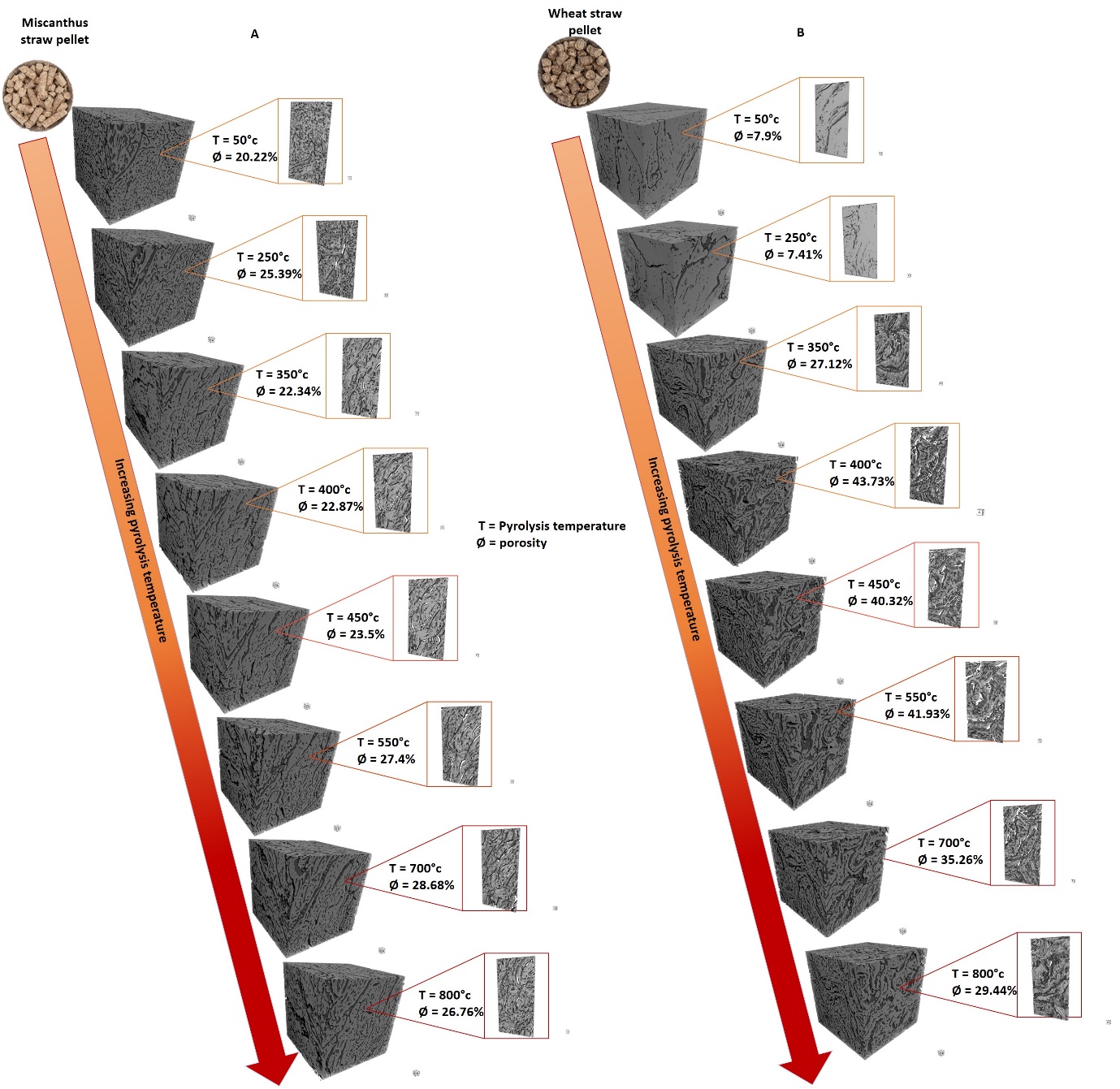


**Supplementary Figure S1: 3D pore morphological view of biochar produced from a) MSP and b) WSP at different pyrolysis temperatures.**

**
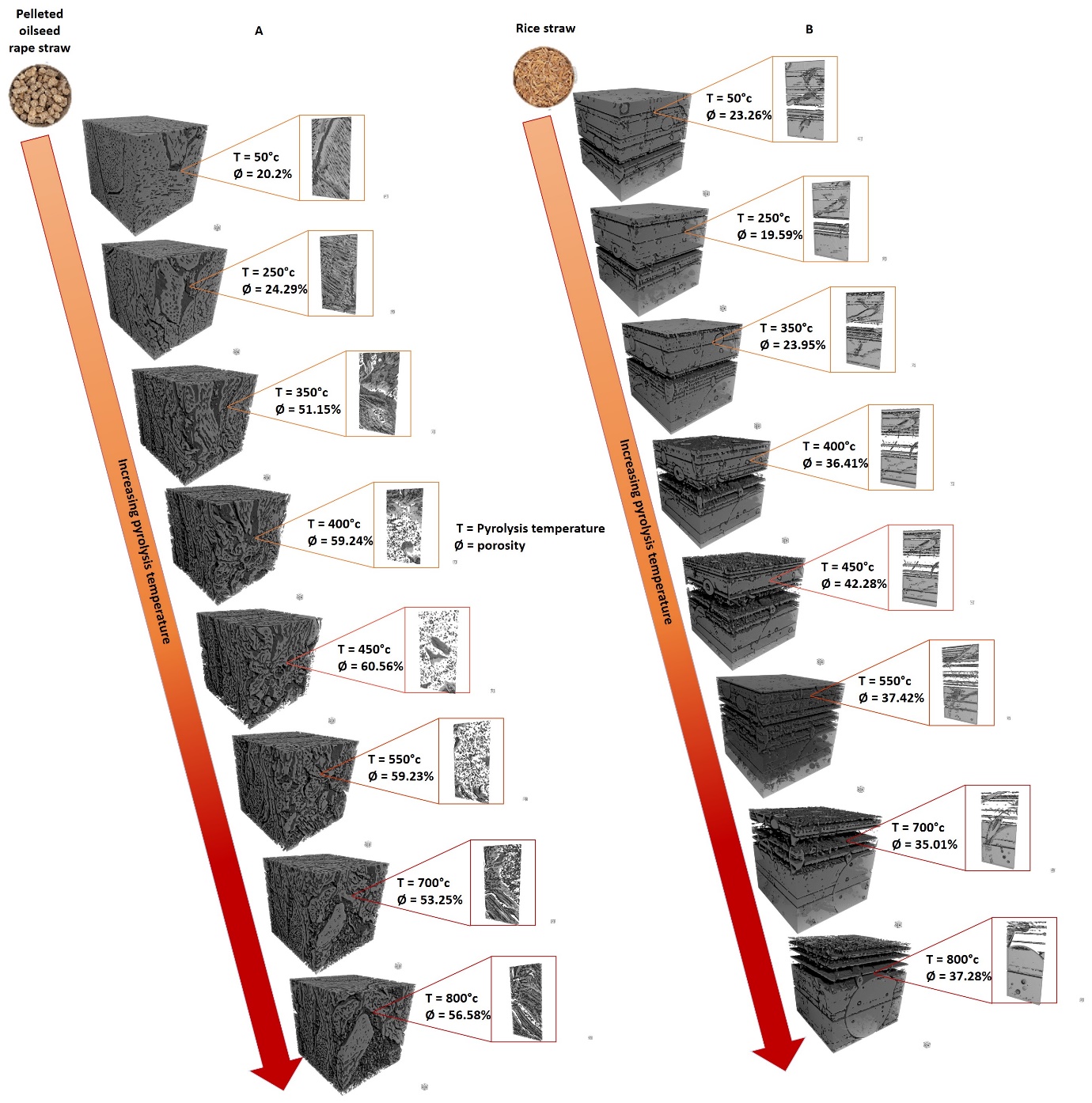
**

**Supplementary Figure S2: 3D pore morphological view of biochar produced from a) OSR and b) RH at different pyrolysis temperatures.**


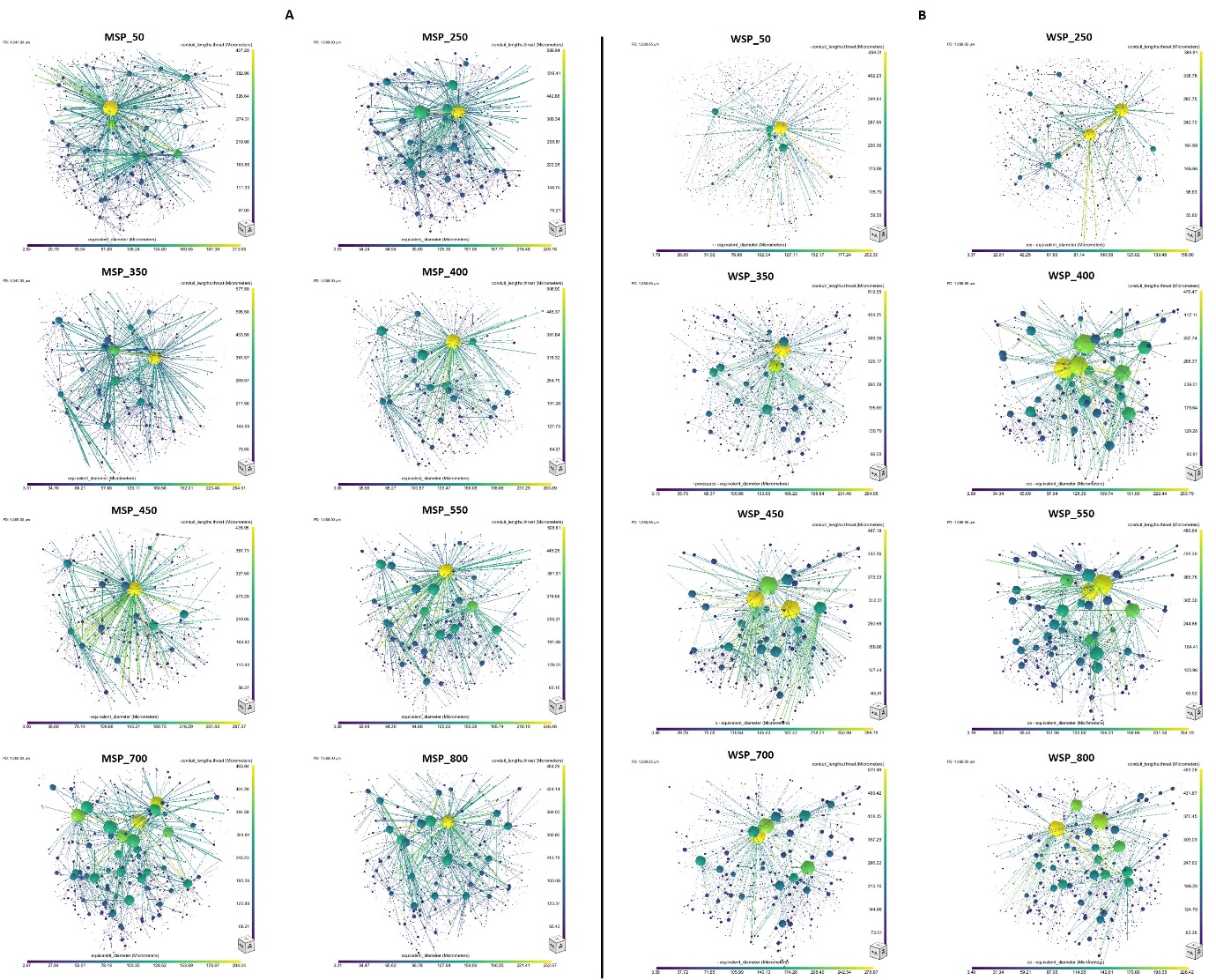


**Supplementary Figure S3: 3D pore network model of biochar produced from a) MSP and b) WSP at different pyrolysis temperatures.**


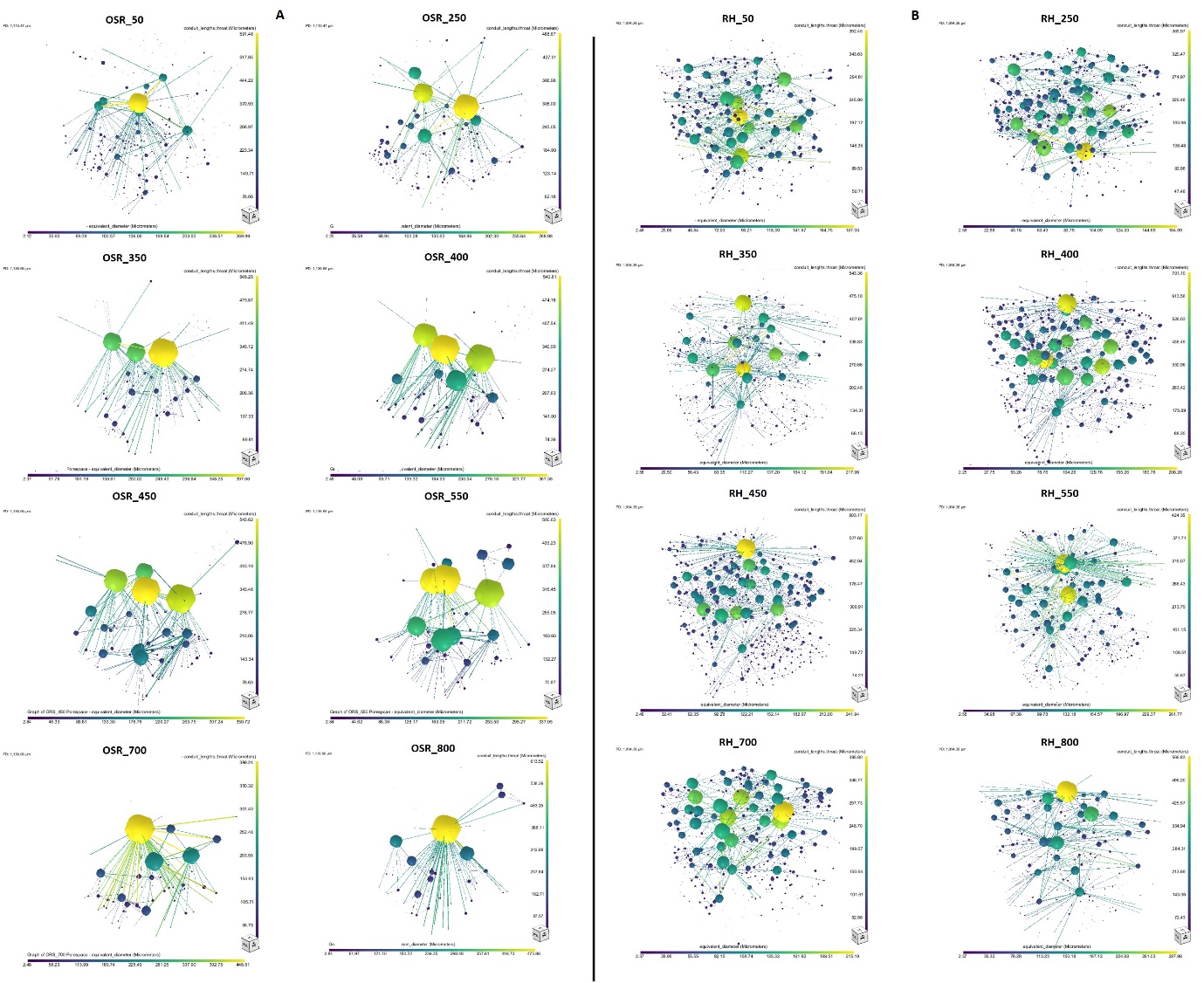


**Supplementary Figure S4: 3D pore network model of biochar produced from a) OSR and b) RH at different pyrolysis temperatures.**

| **Supplementary Table S1: Description of acronyms used for the datasets** | | |
| --- | --- | --- |
| **Feedstock** | **Pyrolysis temperature (°c)** | **Acronyms** |
| Miscanthus straw pellet | 50 | MSP50 |
|  | 250 | MSP250 |
|  | 350 | MSP350 |
|  | 400 | MSP400 |
|  | 450 | MSP450 |
|  | 550 | MSP550 |
|  | 700 | MSP700 |
|  | 800 | MSP800 |
| Wheat straw pellet | 50 | WSP50 |
|  | 250 | WSP250 |
|  | 350 | WSP350 |
|  | 400 | WSP400 |
|  | 450 | WSP450 |
|  | 550 | WSP550 |
|  | 700 | WSP700 |
|  | 800 | WSP800 |
| Oilseed rape straw | 50 | OSR50 |
|  | 250 | OSR250 |
|  | 350 | OSR350 |
|  | 400 | OSR400 |
|  | 450 | OSR450 |
|  | 550 | OSR550 |
|  | 700 | OSR700 |
|  | 800 | OSR800 |
| Rice husk | 50 | RH50 |
|  | 250 | RH250 |
|  | 350 | RH350 |
|  | 400 | RH400 |
|  | 450 | RH450 |
|  | 550 | RH550 |
|  | 700 | RH700 |
|  | 800 | RH800 |


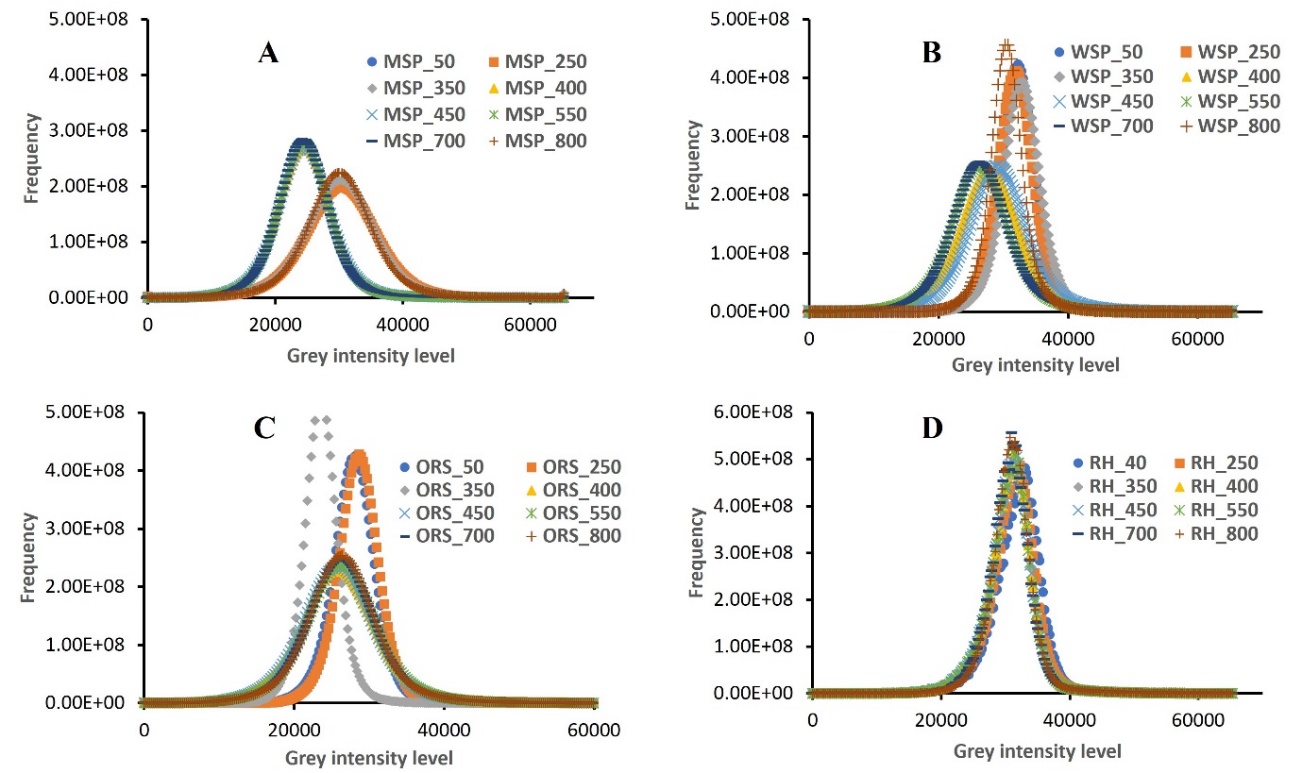


**Supplementary Figure S5: The grayscale distribution for reconstructed 3D dataset of a. MSP, b. WSP, c. OSR, d. RH at all pyrolysis temperature**

**S1. Representative volume determination**

The representative elementary volume (REV) was obtained by successively cropping the image stack for all 4D dataset. To make sure that the features of the particle structure are not affected by the background the first stack was cropped in a manner that only the particle is in the field of view. After the porosity of the first stack was determined, the volume was further reduced consistently for all pyrolysis temperature from 1500X1300X1500 to 500^3^ (Supplementary Figure S6.). to ensure similarity across all 4D data, the cropping was done at similar locations for all the datasets. Using the standard deviation as error bars, 500^3^ was acceptable as REV for all dataset because the porosity was significantly like the original image stack.

**Supplementary Figure S6: porosity of different volume of each biochar**

**S2. Dragonfly workflow for analysing biochar 4D data**

**1. Image Pre-processing**

The total number of slices for each data set is 2160 with a size of 26.3GB. This is too large and would take up a lot of computing time. Before processing, 1000 slices would be selected from the middle of the whole data sets. Further reduction will be done by cropping out the background and selecting a REV following Philip’s procedure.

For developing these workflow, 100 slices and a thin section in the middle were used for a faster computing time. All image processing and analysis were done in dragonfly.

**2. Image Filtering**

Image filter is used to improve segmentation quality, denoise the image and increase contrast between particles and pore spaces. There are a lot of filters in dragonfly (more than 20). After trying all of them, the following three smoothing filters (filter applied to reduce the amount of noise in an image) were considered the closest for biochar data.

1. **Gaussian Filter:** smoothes or blurs an image by preforming a convolution operation with a gaussian filter kernel. The settings used were dimension: 3D; kernel size: 5 and STD: 1.00
2. **Kuwachara filter**: provides non-linear smoothing for adaptive noise reduction. Settings: dimensions – 2D kernel size – 5
3. **Median filter:** smoothes images by replacing each pixel with the median value of its neighbors. Settings: dimension – 3D; kernel size – 5

**
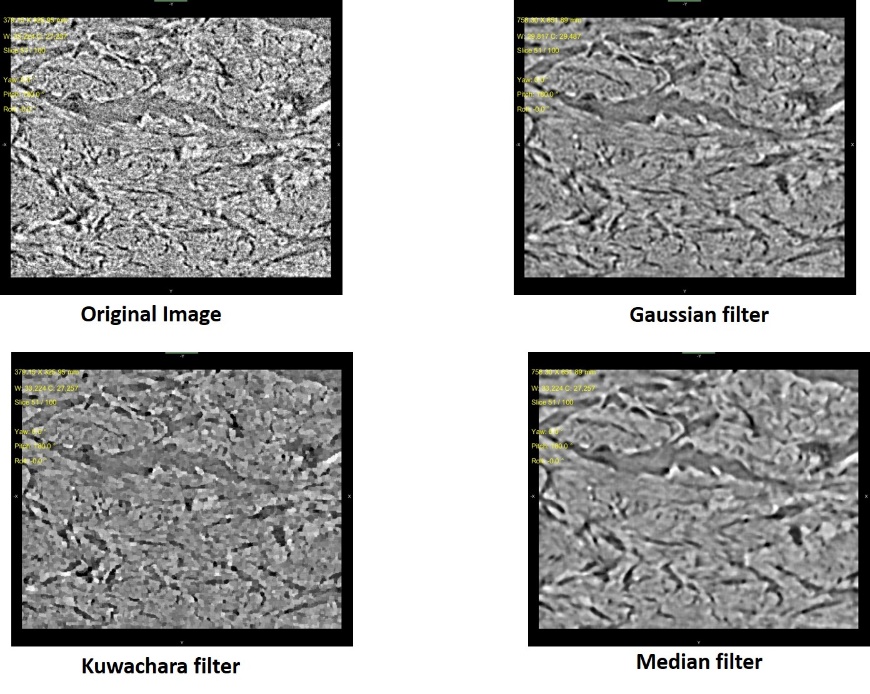
**

**Supplementary Figure S7: XY 2D slice showing different filtering options**

I chose to continue with the median filter because it increased the contrast between particle and pore better while still preserving the edges.

**3. Thresholding**

This is also an image filter which gives an output of an image composed of two basic classes (foreground and background). This is a very important filter for our biochar data because we only need to separate our images into 2 basic classes (foreground – biochar particle and background – porespaces).

Again, dragonfly has a lot of thresholding filters. After trying all of them, 3 of the closest were selected for this presentation.

1. **Isodata:** returns threshold values based on isodata method (i.e. returned thresholds are intensities that separate the image into 2 groups of pixels, where the threshold intensity is midway between the mean intensities of these groups.
2. **Mean:** selects the threshold as the mean of the local greyscale distribution.
3. **Otsu:** threshold clustering algorithm searches for the threshold that minimizes the intra-class variance defined as weighted sum of variances of the two classes.


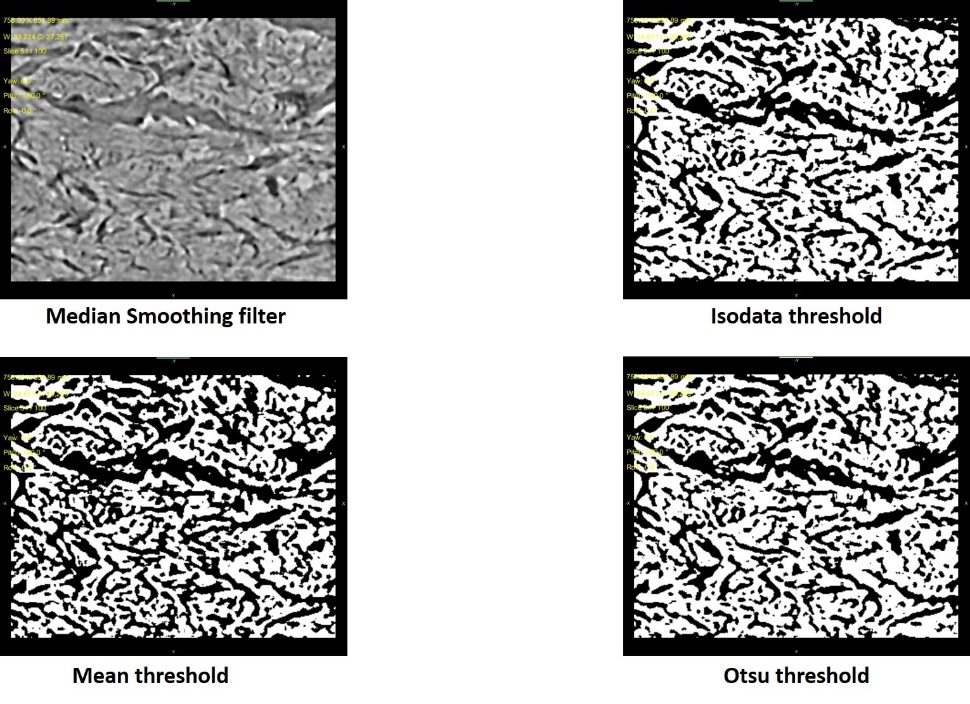


**Supplementary Figure S8: XY 2D slice showing different thresholding options**

I chose Otsu because it gave a more defined threshold that looked more like the original images.


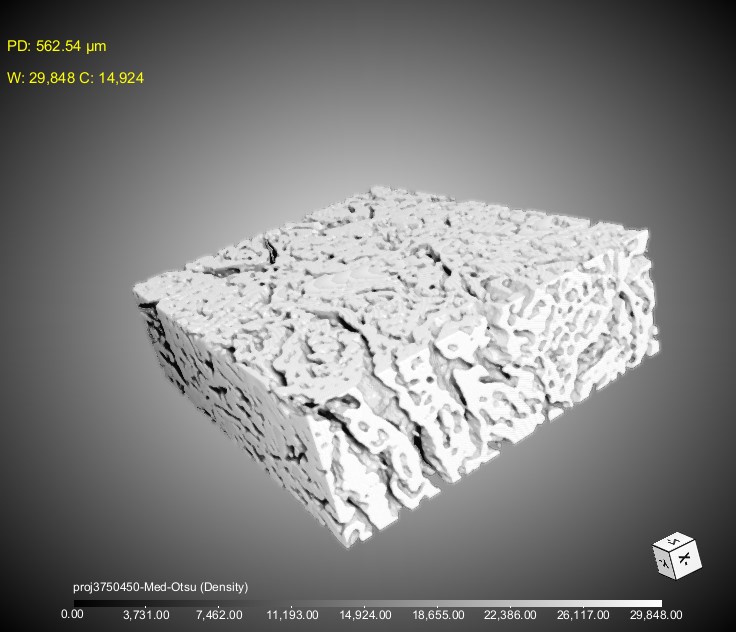


**Supplementary Figure S9: 3D view of the thresholded data with Otsu**

**4. Labelling Pores**

**1. Optimizing watershed segmentation**

The only room where optimization of the watershed segmentation is possible is when choosing the markers/seeds used for the watershed transformation. The markers created from a combination of the distance map and biochar solid ROI can be adjusted to various sizes.

If the size of the markers is too small, the labelled pores are broken into smaller pores (Supplementary Figure S10a). if the size of the markers is however too large, the labelled pores may consist of 2 or more pores classified as one (Supplementary Figure S10b). I tried adjusting the sizes of the markers and produced 2 other different labelled images (Supplementary Figure S10c and d). This did not produce a satisfactory labelling either.


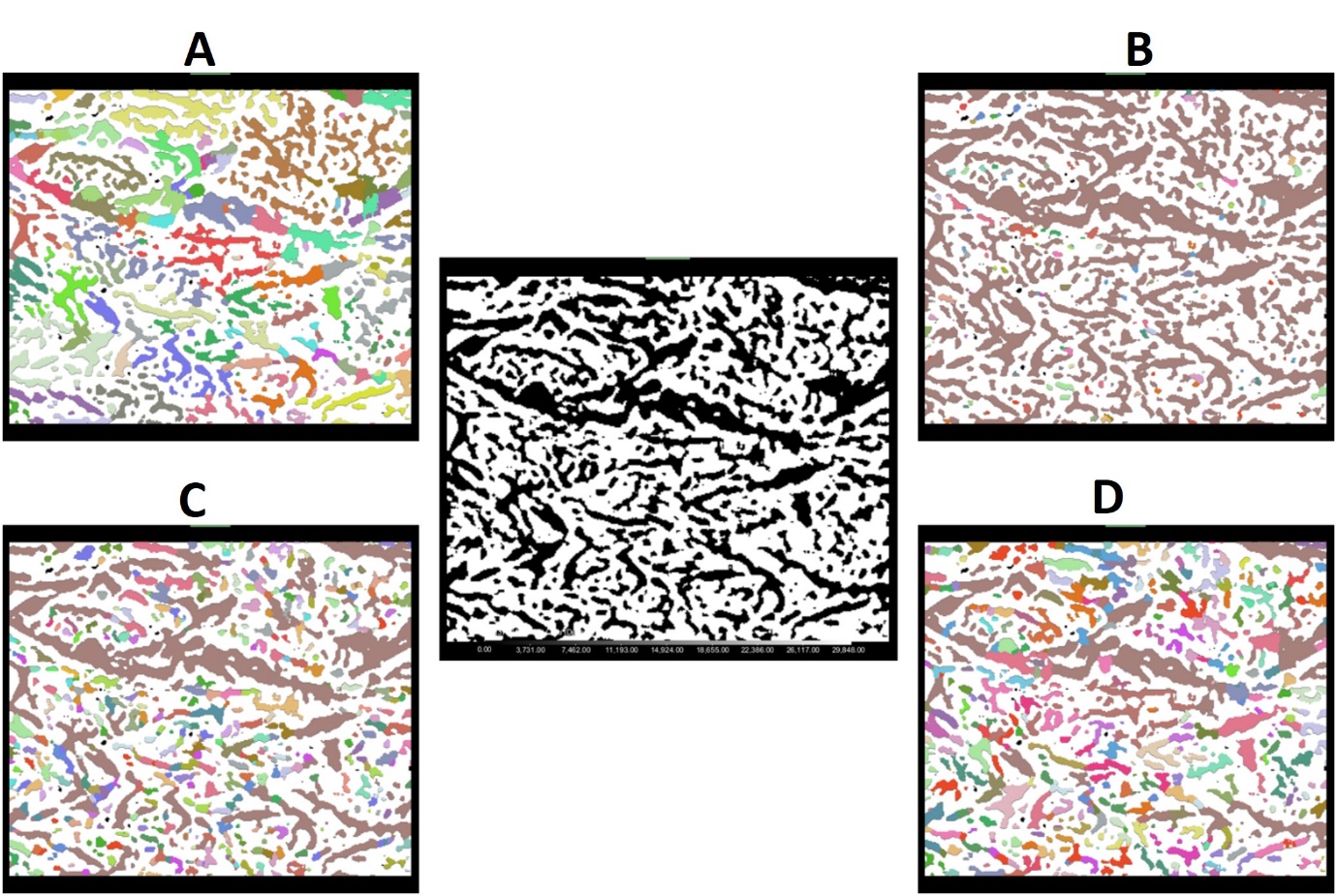


**Supplementary Figure S10: 2D view of labelled images with different marker sizes**

I decided to try the open pore network modelling (openPNM) plugin again. I discarded it in the earlier workflow, but I think it may be a preferred option because:

1. Unlike the watershed segmentation where decision based on markers is subjective, the openPNM has set parameters which can be perfectly replicated across multiple datasets. Choosing openPNM will in some way ensure objectivity of the segmentation process for different datasets thus avoiding operator-dependent bias in the results.
2. The parameters can also be changed to optimize results obtained.

**2. Labelling using the openPNM plugin in dragonfly**

There are 5 parameters using this method that can be adjusted – phase index, sigma, R max, trim isolated pores, and edge tolerance. Supplementary Figure S11 shows a few examples of the 2D view of the images produced from trying numerous combinations of the parameters. Table S2 gives a description of the parameters used in Supplementary Figure S11.


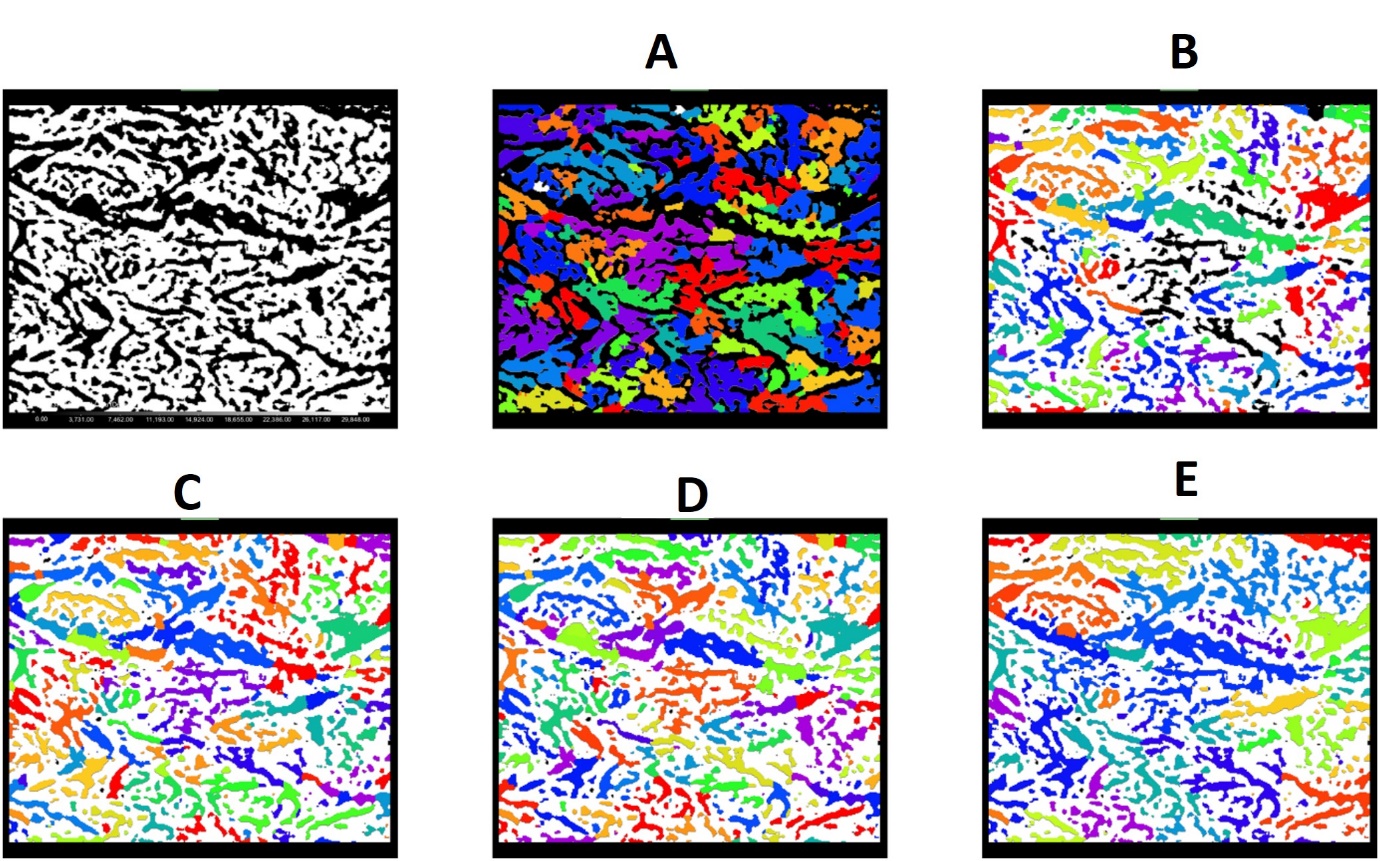


**Supplementary Figure S11: 2D view of labelled images with openPNM**

| **Supplementary Table S2: Parameters used for labelling using OpenPNM** | | | | | |
| --- | --- | --- | --- | --- | --- |
| Figures | Phase index | Sigma | R max | Trim isolated pores | Edge tolerance (%) |
| 2a (default setting) | 0 | 0.4 | 4 | Checked | 0.10 |
| 2b | 1 | 0.4 | 4 | Checked | 0.10 |
| 2c | 1 | 0.4 | 4 | Unchecked | 0.10 |
| 2d | 1 | 0.4 | 4 | Unchecked | 0.50 |
| 2e | 1 | 0.3 | 2 | Unchecked | 0.50 |

The labelling obtained using openPNM were much better than that of the watershed especially looking at Supplementary Figure S11e however, the classification is still not perfect. I decided to try another option of smoothing the thresholding image before segmentation. The process of filtering the Otsu thresholded images was also used by Hyväluoma et al., 2018 for biochar samples.

**3. Filtering Otsu thresholded images before segmentation with openPNM**

Median smoothing filter was applied to the thresholded images using kernel size 5 (Supplementary Figure S12).


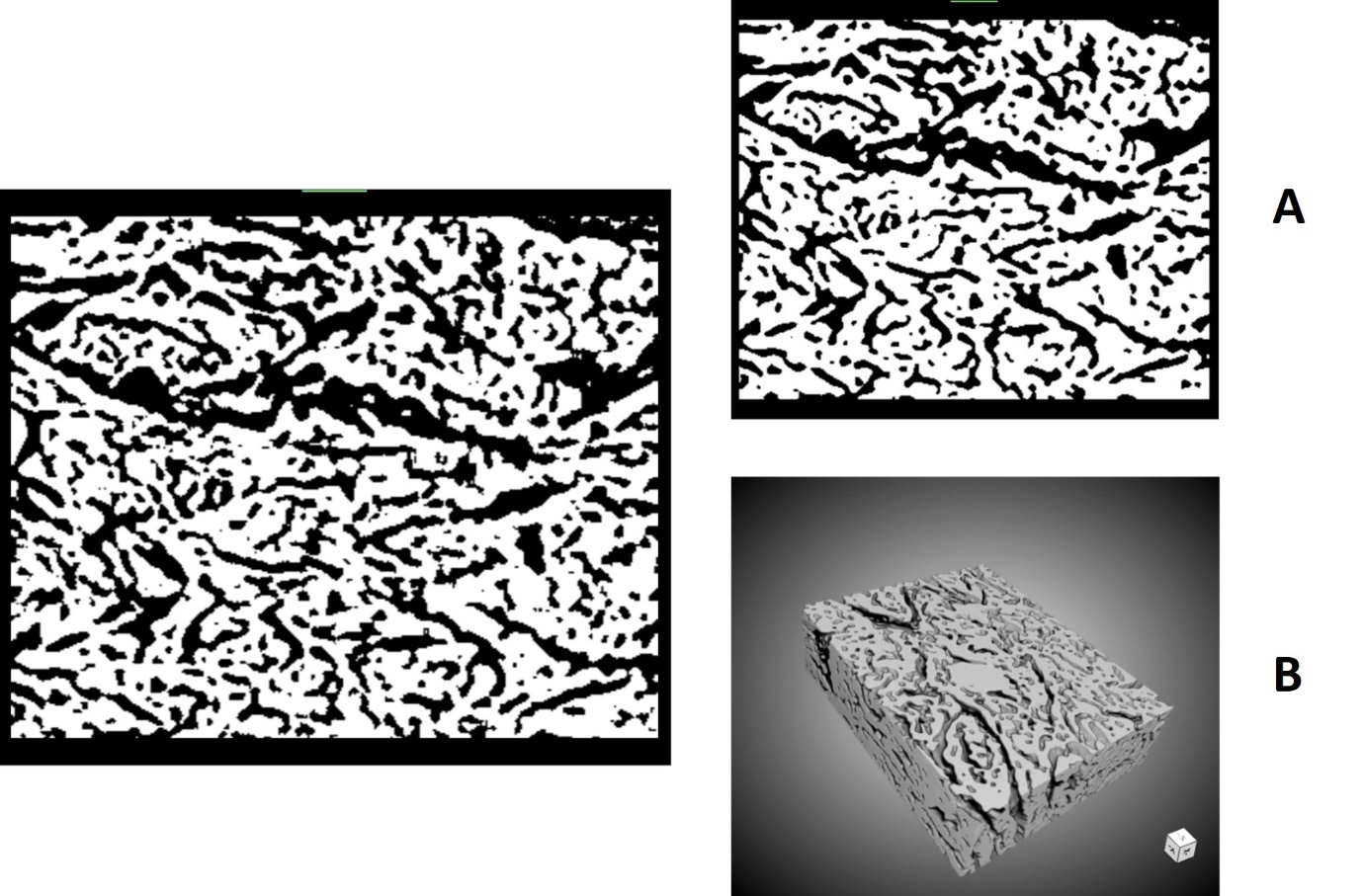


**Supplementary Figure S12: 2d view (a) and 3d view (b) of filtered thresholded image**

A multiROI of the porespace was produced using the openPNM with sigma = 0.1, Rmax = 2, edge tolerance = 0.50% and unchecked trim isolated pores (Supplementary Figure S13).


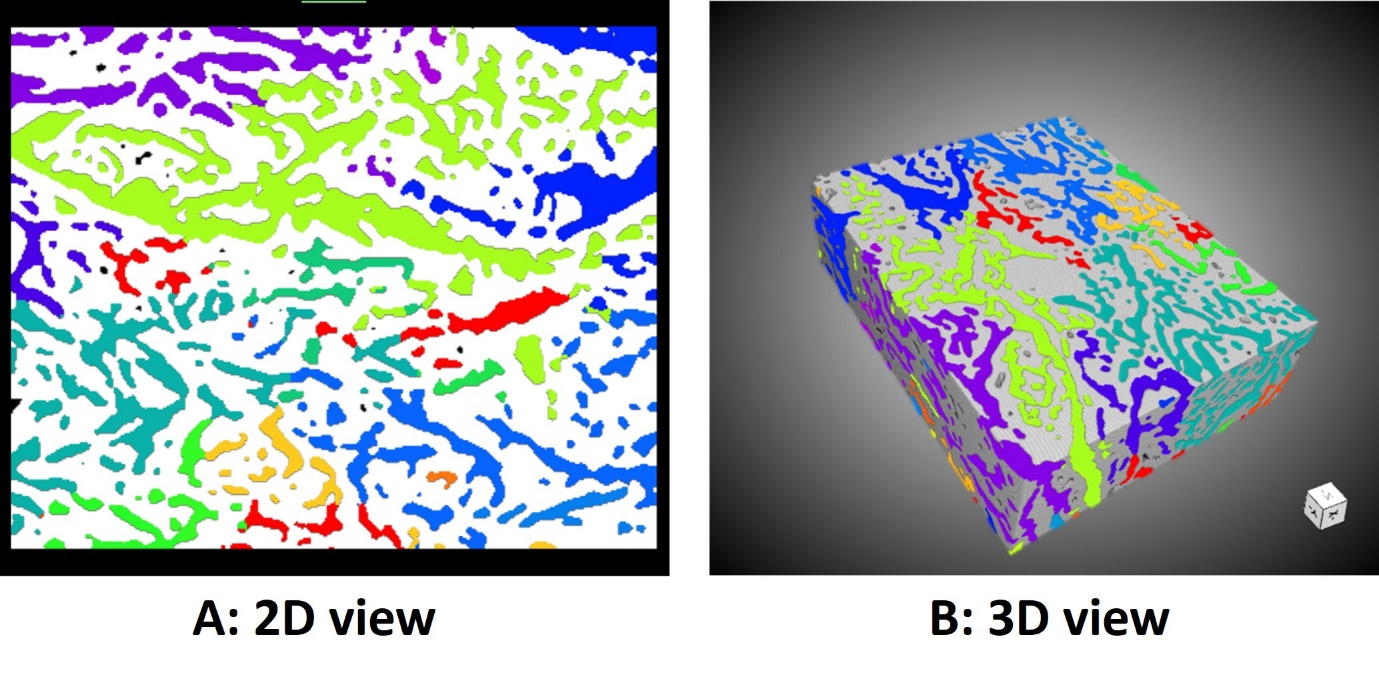


**Supplementary Figure S13: labelled pore spaces**

Looking at the multiROI, some of the pores were not labelled. This can also be confirmed from the porosity. The porosity of the pore ROI before labelling is 34.48% after labelling, the porosity of the pore multiROI is 34.2%. To correct for this, a feature in the dragonfly that allows for creating union, intersects and subtraction of ROI can be used.

1. Get the subtraction of the pore ROI and MultiROI as “a”
2. Get the union of “a” and the multiROI as a new multiROI (Supplementary Figure S14)


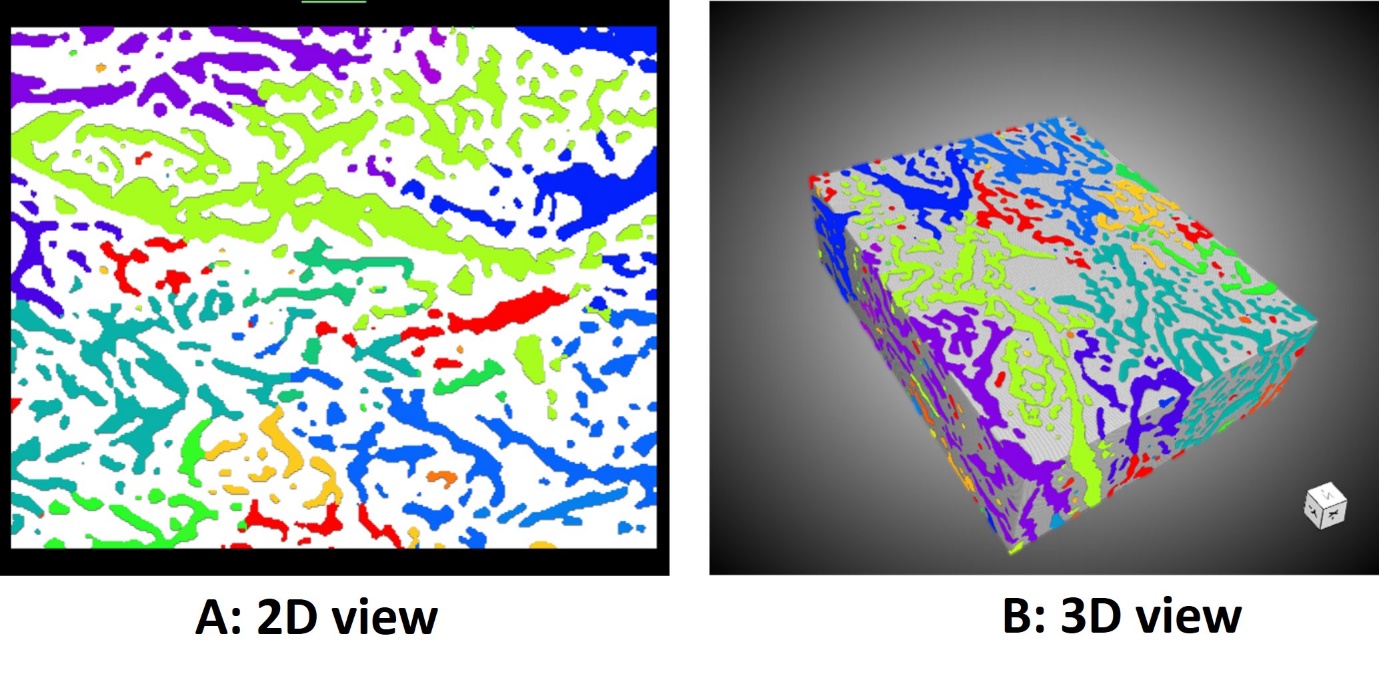


**Supplementary Figure S14: completely labelled pore spaces**

The new multiROI has all the pore spaces labelled and this is also confirmed with the porosity. The porosity of the new multiROI is 34.48% which is the same as the pore ROI.
